# Critical roles of sepsis-reshaped fecal virota in attenuating sepsis severity

**DOI:** 10.3389/fimmu.2022.940935

**Published:** 2022-08-02

**Authors:** Wiwat Chancharoenthana, Nattawut Sutnu, Peerapat Visitchanakun, Vorthon Sawaswong, Suwalak Chitcharoen, Sunchai Payungporn, Alexandra Schuetz, Marcus J. Schultz, Asada Leelahavanichkul

**Affiliations:** ^1^ Tropical Nephrology Research Unit, Department of Clinical Tropical Medicine, Faculty of Tropical Medicine, Mahidol University, Bangkok, Thailand; ^2^ Tropical Immunology and Translational Research Unit, Department of Clinical Tropical Medicine, Faculty of Tropical Medicine, Mahidol University, Bangkok, Thailand; ^3^ Department of Microbiology, Faculty of Medicine, Chulalongkorn University, Bangkok, Thailand; ^4^ Center of Excellence on Translational Research in Inflammatory and Immunology (CETRII), Department of Microbiology, Chulalongkorn University, Bangkok, Thailand; ^5^ Department of Biochemistry, Faculty of Medicine, Chulalongkorn University, Bangkok, Thailand; ^6^ Research Unit of Systems Microbiology, Department of Biochemistry, Faculty of Medicine, Chulalongkorn University, Bangkok, Thailand; ^7^ U.S. Military HIV Research Program, Walter Reed Army Institute of Research, Silver Spring, MD, United States; ^8^ Henry M. Jackson Foundation for the Advancement of Military Medicine, Bethesda , MD, United States; ^9^ Department of Retrovirology, Armed Forces Research Institute of Medical Sciences-United States Component, Bangkok, Thailand; ^10^ Mahidol–Oxford Tropical Medicine Research Unit, Faculty of Tropical Medicine, Mahidol University, Bangkok, Thailand; ^11^ Department of Intensive Care & Laboratory of Experimental Intensive Care and Anesthesiology (L.E.I.C.A), Academic Medical Center, University of Amsterdam, Amsterdam, Netherlands; ^12^ Centre for Tropical Medicine and Global Health, Nuffield Department of Medicine, Oxford University, Oxford, United Kingdom

**Keywords:** sepsis, CLP, microbiome, mycobiome, virome, fecal transplantation

## Abstract

Because studies on all fecal organisms (bacteria, fungi, and viruses) in sepsis are rare and bacteriophages during sepsis might have adapted against gut bacteria with possible pathogenicity, cecal ligation and puncture (CLP; a sepsis mouse model) was evaluated. In fecal bacteriome, sepsis increased *Bacteroides* and Proteobacteria but decreased Firmicutes, while fecal virome demonstrated increased *Podoviridae* when compared with sham feces. There was no difference in the fungal microbiome (predominant Ascomycota in both sham and CLP mice) and the abundance of all organisms between sepsis and control groups. Interestingly, the transfers of feces from CLP mice worsened sepsis severity when compared with sham fecal transplantation, as evaluated by mortality, renal injury (serum creatinine and histology), liver damage (liver enzyme and histology), spleen apoptosis, serum cytokines, endotoxemia, and bacteremia. In contrast, the transfers of fecal viral particles from sepsis mice, but not from sham mice, attenuated inflammation in CLP sepsis possibly through the decrease in several fecal pathogenic bacteria (such as Proteobacteria, Gammaproteobacteria, and Prevotellaceae) as evaluated by fecal microbiome analysis. Perhaps the isolation of favorable bacteriophages in sepsis feces and increased abundance *ex vivo* before oral treatment in a high concentration are beneficial.

## Introduction

Sepsis, an organ failure syndrome produced by a malfunction in the host’s immune system in response to a systemic infection, is one leading cause of death that has been recognized as a major global healthcare issue (1). The gut microbiome is closely associated with sepsis as gut barrier defect can enhance or cause sepsis referred to as ‘gut-origin sepsis’ (2, 3), and severe sepsis also induces intestinal integrity defect (sepsis-induced gut failure) through several mechanisms of intestinal injury (ischemic reperfusion injury, necrosis, and apoptosis) (4). Despite the fact that the pathophysiology of sepsis is complicated and incompletely understood, the disruption of the gut microbiota (gut dysbiosis) predisposes to sepsis may have a negative impact on sepsis outcomes. Indeed, the gut barrier consists of a single layer of epithelial cells, held together by epithelial tight junctions (TJs), with an approximate surface area of 20–30 m^2^, which serves as a selective physical barrier between the host and gastrointestinal (GI) contents, forming the intrinsic mucosal defense system (5). Gut barrier defect (leaky gut) allows the translocation of the microbial molecules (pathogen-associated molecular patterns (PAMPs)) or viable organisms from the gut into blood circulation or other sterile sites of the body (gut translocation) that facilitates systemic inflammation (2). In the gut, bacteria from the phyla Firmicutes (mostly Gram-positive anaerobes) and Bacteroidetes (obligate Gram-negative anaerobes) are the most predominant microbiota (6), followed by fungi, especially *Candida albicans* (in humans) and *Candida* non-*albicans* (in mice), which are the member of the phylum Ascomycota (7). Interestingly, the interactions between bacteria and fungi in the gut are important in sepsis, as fungal administration in mice enhances pathogenic bacteria (8) and antifungal drugs alter the bacterial community (9). Additionally, gut translocation of the major PAMPs of bacteria and fungi, including lipopolysaccharide (LPS) and (1→3)-β-d-glucan from Gram-negative bacteria and *Candida* spp., respectively, is partly a factor modifying sepsis severity, and control of the abundance of these organisms in the gut might attenuate sepsis (2).

Although the impact of bacteria and fungi in the gut on sepsis pathogenesis has been profoundly studied (10, 11), data on the whole gut microbiome, including the bacteriome, the virome, and mycobiome, in sepsis remains ill-defined. Bacteriophages (phages), the virus that infects and replicates within bacteria, have been proposed to be used against several antibiotic-resistant bacteria as adjunctive therapy (12, 13). Due to the specificity of phages against only limited bacterial strains, partly from the strain-specific molecules for the entry of viruses, the combination of phages is used to overcome this limitation (14). Because i) phages are a natural bacterial control in the ecosystem (15, 16), including the intestines (17, 18), and ii) sepsis (and systemic inflammation) alters gut microbiota (dysbiosis) and increases harmful microorganisms in the gut (19, 20), the increased bacteria with possible pathogenicity in the host with sepsis might naturally induce phage propagation, and these bacteria might partly be controlled by the induced phages. Thus, the adaptation of phages in the host might already be a combination of the necessary phages for the specific conditions, and the boost-up of these phage combinations might be beneficial as effective phages for individual patients.

Our goals were to investigate fecal organisms using a sepsis mouse model in order to test the following possibilities: i) sepsis may interfere with all gut organisms (bacteria, fungi, and viruses), ii) balanced homeostasis may exist in the sepsis intestine, and iii) gut microbial modification by the transfer of feces from normal mice (sham feces) or phages (viral particles) from sepsis mice may attenuate sepsis. Hence, the cecal ligation and puncture (CLP) sepsis mouse model was used for the experiments.

## Materials and methods

### Animals

Male 8-week-old C57BL/6 mice were purchased from Siam Nomura (Samut Sakhon, Thailand) and had free access to water and chow before and after surgery. For sepsis induction, CLP was performed according to previous publications (21–24). Briefly, the cecum was ligated at 12 mm from the cecal tip, punctured twice with a 21-gauge needle, and gently pressed to express a small amount of fecal material before being placed into the abdominal cavity. In sham mice, the cecum was only identified through abdominal incision before suturing layer by layer with 6-0 nylon sutures. Then, 1 ml of prewarmed normal saline solution (NSS) with tramadol at 25 mg/kg/dose was subcutaneously administered after surgery, at 6 and 18 h post-CLP. Fecal samples for microbiome analysis were collected at 24 h of surgery (sham and CLP) after mice were sacrificed by cardiac puncture under isoflurane anesthesia.

### Fecal analysis for bacteriome and mycobiome

Fecal samples from each mouse (0.25 g per mouse) from different cages in each experimental group were collected for microbiota analysis following a previous protocol (22, 25–28). In short, metagenomic DNA was extracted by DNeasy PowerSoil Kit (Qiagen, Maryland, USA) using the Universal prokaryotic 515F (forward, (5′-GTGCCAGCMGCCGCGGTAA-3′) and 806R (reverse, 5′-GGACTACHVGGGTWTCTAAT-3′), with the Illumina adapter and Golay barcode sequences for 16S rRNA gene V4 library construction in Miseq300 platform (Illumina, San Diego, CA, USA). The raw sequences and operational taxonomic unit (OTU) were classified following Mothur’s standard operating platform. In parallel, to identify the taxonomic profiles of fungal microbiota (mycobiome) in feces, the DNA was extracted from fecal samples. The universal eukaryotic primers ITS3 (forward; 5′-GCATCGATGAAGAACGCAGC-3′) and ITS4 (reverse; 5′-TCCTCCGCTTATTGATATGC-3′) were used for identifying the gut mycobiota. The fungal DNA library was sequenced using the Miseq system (Illumina, San Diego, CA, USA) at Omics Sciences and Bioinformatics Center, Chulalongkorn University. Forward and reverse primers were removed from raw sequences using cutadapt version 1.18 and trimmomatic version 0.39 with the sliding window option to trim individual sequences where the average quality scores are less than 15 across four base pairs. To identify the composition of fungi in fecal samples, amplicon sequence variants (ASVs) were analyzed using the QIIME2 plugin DADA2 pipeline. Unclassified phylum or higher fungal classification was removed. To confirm non-fungal origin, the fungal classification was analyzed by a BLAST-based tool (https://blast.ncbi.nlm.nih.gov) (National Center for Biotechnology Information, Bethesda, MD, USA).

### Fecal virome and quantitative analysis (fluorescent staining) of viral particles in feces

The procedure of virome sample processing was carried out following a previous study (29), with some modifications. Briefly, 50 mg of fecal sample was resuspended in 1 ml of phosphate-buffered saline (PBS) and centrifuged at 8,000 × *g* at 4°C for 5 min. The supernatant was filtered through a 0.45-µm syringe filter (Sartorius, Göttingen, Germany). The filtrate at 500 µl was then treated by a nuclease cocktail containing 30 U of RNase One (Promega, Madison, WI, USA), 3 U of Baseline-ZERO (Epicentre, Madison, WI, USA), 30 U of Benzonase (Novagen, Darmstadt, Germany), and 14 U of Turbo DNase (Ambion, Thermo Fisher Scientific, Waltham, MA, USA) in 1× Turbo DNase buffer. The sample was incubated at 37°C for 1.5 h and was extracted for viral nucleic acid using the MagMAX™ Viral RNA Isolation kit (Applied Biosystems, Thermo Fisher Scientific, USA). The cDNA was constructed based on the RevertAid First Strand cDNA Synthesis Kit (Thermo Fisher, USA) with 100 pmol of Sol A random primer: 5′-GTT TCC CAC TGG AGG ATA NNN NNN NNN-3′ in 20 µl of reaction following the manufacturer’s protocol. After the first strand synthesis was done, 5 U of Klenow Fragment DNA polymerase (New England Biolabs, Ipswich, MA, USA) was added to the reaction and incubated at 37°C for 60 min and 75°C for 20 min. The random amplification of cDNA was performed based on a PCR comprising 2 µM of Sol B primer: 5′-GTT TCC CAC TGG AGG ATA-3′, 0.2 U of Phusion DNA polymerase (Thermo Fisher Scientific, USA), 0.25 of mM of dNTPs, 5 µl of cDNA template, and 1× Phusion HF buffer. The thermal profile was 95°C for 5 min, 5 cycles of 95°C for 30 s, 59°C for 60 s, and 72°C for 90 s, 35 cycles of 95°C for 30 s, 59°C for 30 s, and 72°C for 90 s (+2 s per cycle) followed by 72°C for 10 min and hold at 4°C. The random amplified products were then used for DNA library preparation using NEBNext Ultra II DNA Library Prep Kit for Illumina (New England Biolabs, USA). Finally, the library was paired-end sequenced (2 × 250 cycles) using the Illumina MiSeq sequencing platform with 10% PhiX spike-in. The raw FASTQ reads were quality checked by FastQC. Then, the low-quality sequences (<Q20) and adaptor sequences were trimmed by Trimmomatic (version 0.36) (30). The host reads were filtered out by mapping the reads against the reference mouse genome (GRCm38) using Bowtie2 (31). The unmapped reads were *de novo* assembled by EnsembleAssembler (32). The contigs were BLASTx search against viral protein database (collected from ftp://ftp.ncbi.nih.gov/refseq/release/viral/) using with e−10 E−value cutoff. The taxonomically classified contigs were subsequently used as the reference sequence for mapping the viral reads to count the hits of each viral taxon. For quantitation of fecal viruses, the filtrated samples were diluted and stained with SYBR Gold (Thermo Fisher, CA) for DNA viruses and SYBR Green II (Solarbio, Beijing, China) for RNA viruses for 15 min before washing as previously described (33–35) and visualizing by LSM 800 Airyscan confocal laser scanning microscopy (CLSM; Carl Zeiss, Jena, Germany).

### Fecal transplantation before sepsis surgical induction

To determine the impact of fecal organisms on the CLP model, fecal transfers (fecal transplantation) were performed following a modified protocol (36). Briefly, fecal samples from cecum and colon in mice after 24 h post-CLP or post-sham were collected, diluted with PBS in a ratio of 1 g feces:0.5 ml PBS, and thoroughly mixed. Notably, colon fecal contents in sham mice need to be minced before being mixed with PBS, while CLP stools were usually liquified at 24 h post-surgery. After that, the solid fractions were separated by centrifugation at 13,000 × *g* for 10 min (at 4°C), and the fresh supernatant at 10 ml/kg was orally administered to the recipient mice once daily for 5 days. At 4 h after the last dose of fecal gavage, CLP was performed in the recipient mice as previously mentioned. Sham surgery was performed only in mice with fecal gavage by CLP feces, but not the gavage by sham feces, because all parameters of sham with fecal transplantation (sham feces or CLP feces) were not different from sham without fecal gavage (data not shown). Because the worsening of sepsis after transfers of sepsis feces might be due to fecal viruses, oral gavage of viral particles separated from sham and CLP mice after 24 h of surgery was also conducted. For the extraction of viruses, fecal samples from the cecum and colon were mixed with SM buffer (150 mM of NaCl, Tris-HCl pH 6.5, 10 mM of MgCl_2_, and 1 mM of CaCl_2_). The samples were centrifuged at 1,000 × *g* for 1 min to remove the tissue, followed by centrifugation at 5,000 × *g* for 30 min to separate the solid part.. The phage supernatants were collected and filtered through 0.45-µm pore-size syringe filters. All filtrated samples for viruses that were freshly extracted from the feces of one CLP mouse were transferred to one recipient mouse, and the process was repeated once daily for 5 days. Then, CLP or sham was performed for 4 h after the last dose of the phage administration.

### Transfer of viral particles before sepsis surgical induction

Because the impact of crude fecal transplantation, similar to the administration of all fecal organisms (bacteria, virus, and fungi), could differ from the fecal transfer of viral particles (partly bacteriophages or phages) alone, viral particles freshly isolated from mice were orally administered thrice a day for 5 days prior to CLP (or sham) surgery. For the phage isolation, fecal samples from the cecum and colon were mixed with SM buffer (150 mM of NaCl, Tris-HCl pH 6.5, 10 mM of MgCl_2_, and 1 mM of CaCl_2_) before centrifugation at 1,000 × *g* for 1 min to remove the tissue, followed by a 5,000 × *g* centrifugation for 30 min to precipitate the bacterial cell. Then, the supernatants containing phages were collected and filtered through 0.45-µm syringe filters. Total feces per mouse after sham and CLP surgery with the approximate weight of 0.7 ± 0.3 and 0.4 ± 0.2 g, respectively, was diluted by 2 and 1.5 ml of SM buffer, respectively. The fecal samples of sham mice required a higher volume of dilution because of the well-formed consistency of the feces, while CLP fecal samples were liquefied for easier phage preparation. The abundance of viral particles in the samples was determined by staining with SYBR Gold (Thermo Fisher) and SYBR Green II (Solarbio, China) for 15 min before washing and visualizing by LSM 800 Airyscan CLSM (Carl Zeiss, Jena, Germany) as previously described (33–35). The total phage preparation from a mouse was isolated daily and divided into three times of oral administrations (keeping the preparations at 4°C between the doses). Then, the preparations at 0.5 × 10^9^ viral particles per dose (approximately 10 ml/kg/dose) were orally administered every 4 h for one recipient mouse per day. After 5 days of administration, CLP surgery was performed the next day as described above.

### Mouse blood sample analysis and gut leakage measurement

At 24 h post-surgery, all mice were sacrificed through cardiac puncture under isoflurane anesthesia with sample collection (blood and organs). Kidney injury was determined by blood urea nitrogen (BUN) and serum creatinine using QuantiChrom (DIUR-500 and DICT-500, respectively) (BioAssay, Hayward, CA, USA). Liver damage and serum cytokines were determined by EnzyChrom Alanine Transaminase assay (EALT-100) (BioAssay) and enzyme-linked immunosorbent assays (ELISA) for mouse cytokines (Invitrogen, Carlsbad, CA, USA), respectively. In addition, blood leukocyte determination was performed by mixing blood with 3% acetic acid, a hemolytic solution, with a ratio of blood: acetic acid at 6:100 by volume before counting with a hemocytometer. In parallel, the Wright-stained blood smear was examined for the percentage of polymorphonuclear cells (PMNs) and lymphocytes, and the total cell numbers were calculated by the total count from the hemocytometer multiplied by the percentage of cells from the Wright-stained slide. Serum endotoxin (LPS) was measured by HEK-Blue LPS Detection (*In vivo*Gen, San Diego, CA, USA), and the data were recorded as 0 when LPS values were less than 0.01 EU/ml because of the limited lower range of the standard curve. For bacterial burdens in blood, mouse blood samples in several dilutions were directly spread onto blood agar plates (Oxoid, Basingstoke, Hampshire, UK) and incubated at 37°C for 24 h before the enumeration of bacterial colonies. Gut permeability was determined by fluorescein isothiocyanate (FITC)-dextran assay (20, 37) using an oral administration of FITC-dextran, a non-absorbable molecule with 4.4-kDa molecular mass (Sigma-Aldrich, St. Louis, MO, USA), at 12.5 mg at 3 h before the detection in serum by fluorospectrometer (NanoDrop 3300; Thermo Fisher Scientific, Wilmington, DE, USA).

### Histological analysis and immunohistochemistry imaging

The semi-quantitative evaluation of renal histology on paraffin-embedded slides was performed after 10% neutral buffered formalin fixation, followed by hematoxylin and eosin (H&E) staining at ×200 magnification in 10 randomly selected fields for each mouse as previously described (26, 38). Briefly, the renal injury was defined as tubular epithelial swelling, loss of brush border, vacuolar degeneration, necrotic tubules, cast formation, and desquamation using the following scoring method: 0, area of damage <5%; 1, area of damage 5%–10%; 2, area of damage 10%–25%; 3, area of damage 25%–50%; and 4, area of damage >50%. In parallel, an anti-active caspase-3 antibody (Cell Signaling Technology, Beverly, MA, USA) was used for immunohistochemistry staining on 4-m paraffin sections, which were then evaluated in 10 randomly selected ×200 magnified fields as previously described (20). The spleen apoptosis score was expressed as positive cells per high-power field. However, the liver histological score was the sum of hepatocyte injury characteristics (cytoplasmic color fading, vacuolization, nuclear condensation, nuclear fragmentation, nuclear fading, and erythrocyte stasis) ranging from 0 to 5 multiplied by grades of damage of 0 = no, 1 = mild, 2 = moderate, and 3 = severe, following a publication (39).

### Statistical analysis

Mean ± standard error (SE) was used for data presentation. The differences between groups were examined for statistical significance by one-way analysis of variance (ANOVA) followed by Tukey’s analysis or Student’s T-test for multiple and two-group comparisons, respectively. The time-point experiments and survival analysis were analyzed by the repeated-measures ANOVA and the log-rank test, respectively. All statistical analyses were performed with SPSS 11.5 software (SPSS, IL, USA) and GraphPad Prism version 9.3.1 software (La Jolla, CA, USA). A *p*-value of <0.05 was considered statistically significant.

## Results

### Alteration of organism in feces of mice after 24 h of cecal ligation and puncture sepsis as compared with sham mice

**Figure 1:**
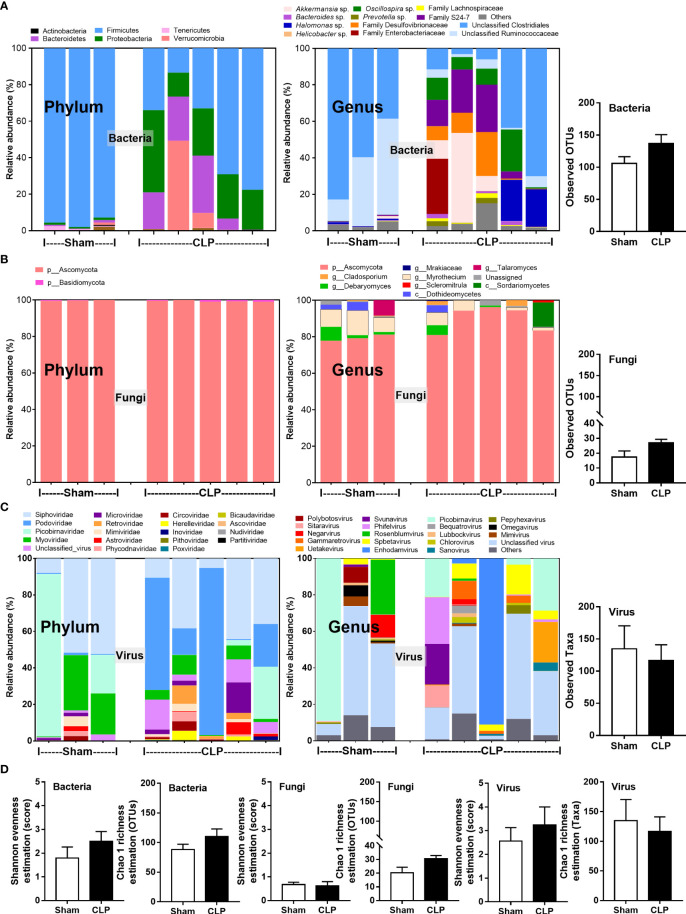
The fecal abundance of microbiome analysis on 16S rRNA (bacteria) **(A)** and nuclear ribosomal internal transcribed spacer (ITS) (fungi) **(B)** or random sequence (viruses) **(C)** from feces of mice after 24 h of sham or cecal ligation and puncture (CLP) surgery in phylum level and genus level together with the observed operational taxonomy units (OTUs) of bacterial and fungal analysis and observed taxa of virome analysis are demonstrated. Alpha diversity (Shannon and Chao-1 analysis) of bacteriome, mycobiome, and virome **(D)** are also indicated.

**Figure 2:**
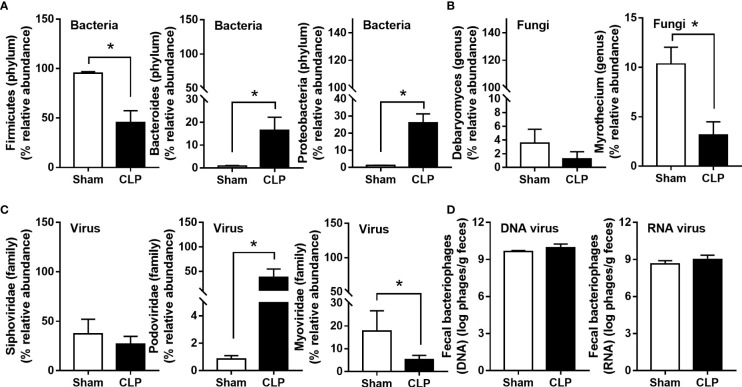
The fecal abundance of bacteria (Firmicutes, *Bacteroides*, and Proteobacteria) **(A)**, fungi (*Debaryomyces* and *Myrothecium*) **(B)**, and viruses (*Siphoviridae*, *Podoviridae*, and *Myoviridae*) **(C)** with quantitative analysis on DNA and RNA viruses by fluorescent staining **(D)** from feces of mice after 24 h of sham or cecal ligation and puncture (CLP) surgery are demonstrated (n = 3 in sham and 5 in CLP). *, *p* < 0.05 between the indicated group.

Microbiome analysis from feces of mice after 24-h surgery demonstrated the prominent differences in bacteria and viruses, but not fungi, when compared between sham and CLP mice **(** **Figures 1A–D** **)**. In fecal bacteriome, Firmicutes (the most prominent bacteria in a healthy host with possible beneficial effects) (40) was predominant in sham mice, while Proteobacteria (the Gram-negative aerobes and facultative anaerobes) (8) and *Bacteroides* (the most prominent Gram-negative anaerobes with possible pathogenesis in some conditions) (25) were predominant in CLP mice **(** **Figure 1A**, left, and **Figure 2A** **)**. Despite no differences in the observed OTUs (the operational definition used to classify groups of closely related individuals) and the microbial diversity (Shannon and Chao-1), several Proteobacteria bacteria, including *Enterobacter* spp., *Desulfovibrio* spp., *Oscillospira* spp., and *Halomonas* spp., were identified in CLP mice **(** **Figure 1A**, middle and right, and **Figure 1D** **)**. Notably, the similar OTUs between sham and CLP mice suggested a comparable number of bacterial species between these groups, which were supported by the representative microbial diversity score (Shannon and Chao-1) (41). For fecal mycobiome (fecal fungi), there was a subtle alteration in sham and CLP mice in all parameters **(** **Figure 1B** **)**, partly due to the less abundance of fecal fungi in mice than in humans (19, 42, 43). Although Ascomycota (the phylum of *C. albicans*, the most prominent fungi in the human gut) was not different between sham and CLP mice, the fecal abundance of *Myrothecium*, a genus of fungi in the Ascomycota group with a limited report of infection (44), in CLP mice was less than that in sham mice **(** **Figure 2B** **)**. In the fecal virome, the predominant viruses in sham mice were *Siphoviridae* (double-stranded DNA phages against some *Lactobacilli* spp.) (45) and *Picobirnaviridae* (the double-stranded RNA phages against several bacteria that could cause diarrhea) (46), and in CLP mice, the viruses were *Siphoviridae* and *Podoviridae* (the double-stranded DNA phages against some *Salmonella* spp.) (47) **(** **Figures 1C**, **2C** **)**. Meanwhile, *Myoviridae* (the double-stranded DNA phages against some *Enterobacteria*) (48) in sham mice was higher than in CLP mice **(** **Figures 1C**, **2D** **)**. Despite a limitation of random sequencing of virome on an analysis of the viral abundance in feces, direct fluorescent staining of viruses (DNA and RNA viruses) was not different between CLP and sham mice **(** **Figure 2C** **)**.

### Fecal transplantation of feces from cecal ligation and puncture mice worsened sepsis in recipient mice

**Figure 3:**
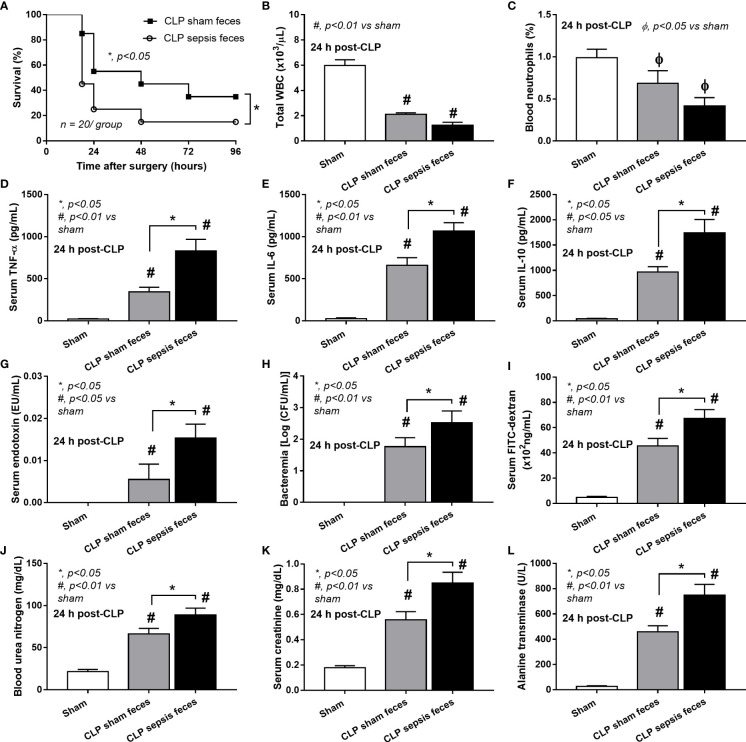
Characteristics of cecal ligation and puncture (CLP) mice with fecal transplantation of sham feces or CLP feces or sham mice with CLP feces (Sham) as evaluated by survival analysis **(A)**, peripheral blood leukocytes and neutrophils **(B, C)**, serum cytokines (TNF-α, IL-6, and IL-10) **(D–F)**, serum endotoxin **(G)**, bacteremia **(H)**, gut leakage (FITC-dextran) **(I)**, renal injury (blood urea nitrogen and serum creatinine) **(J, K)**, and liver enzyme (serum alanine transaminase) **(L)** are demonstrated (n = 6–8 per group). *, *p* < 0.05 between the indicated group; #, *p* < 0.01 vs sham.

**Figure 4:**
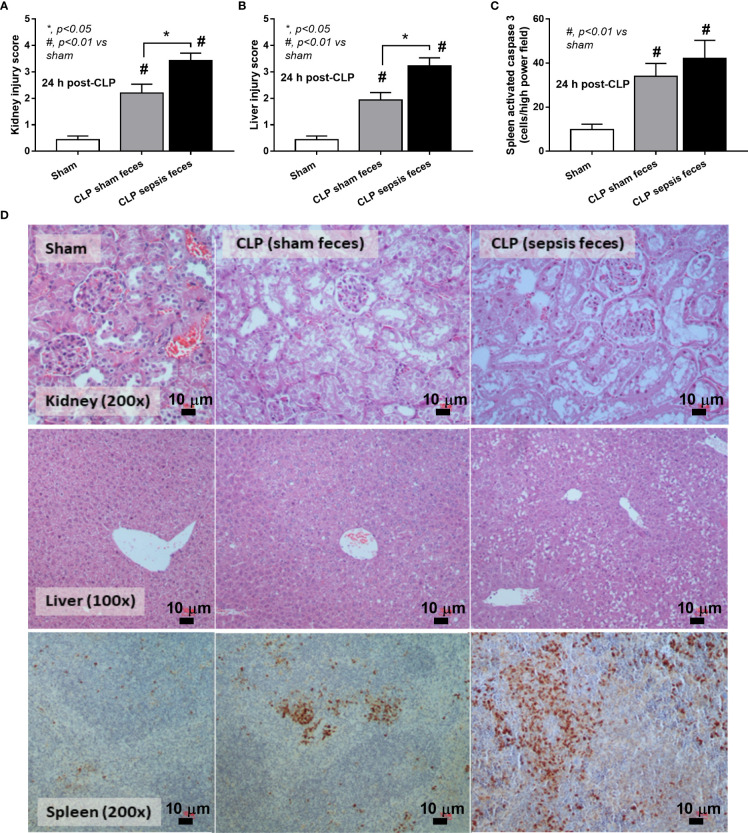
Characteristics of cecal ligation and puncture (CLP) mice with fecal transplantation of sham feces or CLP feces or sham mice with CLP feces (Sham) as evaluated by a histological score of kidney and liver **(A, B)** and spleen apoptosis **(C)** with the representative pictures **(D)** (original magnification ×200) of hematoxylin and eosin (H&E) staining (kidney and liver) and activated caspase 3-stained apoptosis from spleen are demonstrated (n = 6–8 per group). *, *p* < 0.05 between the indicated group; #, *p* < 0.01 *vs* sham.

**Figure 5:**
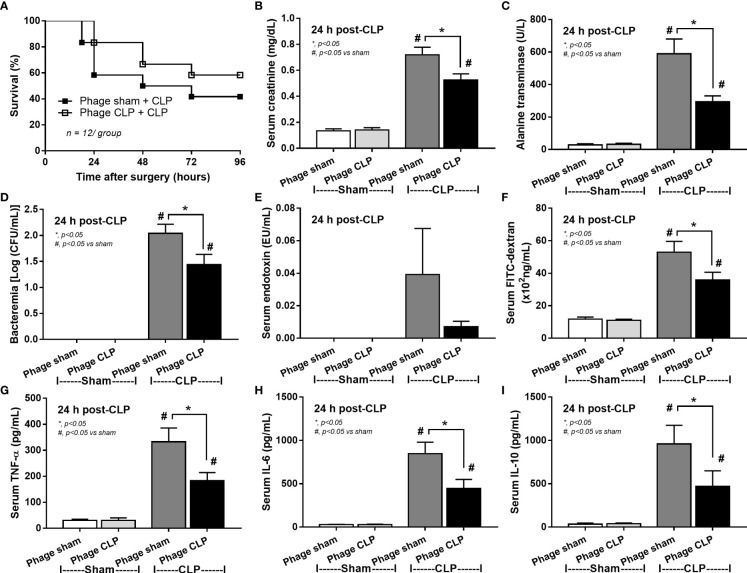
Characteristics of cecal ligation and puncture (CLP) mice with transfers of viral particles that were isolated from sham feces (phage sham) or CLP feces (phage CLP) before performing sham or CLP surgery as evaluated by survival analysis **(A)**, serum creatinine **(B)**, serum alanine transaminase **(C)**, bacteremia **(D)**, endotoxemia **(E)**, gut barrier defect (FITC-dextran assay) **(F)**, and serum cytokines (TNF-α, IL-6, and IL-10) **(G–I)** are demonstrated (n = 12 per group for A and 8–10/group for B–I). *, *p* < 0.05 between the indicated group; #, *p* < 0.05 vs sham.

Although it has been previously mentioned that the transfers of feces from healthy and sepsis mice attenuate and worsen sepsis, respectively (49–51), the studies using the transfers of fresh feces are still very limited, and the conclusions are still inconsistent. Here, daily fecal gavage from CLP mice for 5 days before CLP surgery worsened sepsis severity as compared with CLP operation on sham fecal transfer recipients. Accordingly, CLP in mice with oral gavage by sepsis feces demonstrated a higher mortality rate, serum cytokines, endotoxin, bacteremia, gut leakage (FITC-dextran), and organ injury (liver and kidney) when compared with CLP with sham feces, despite the non-difference in spleen apoptosis **(** **Figures 3A–L**, **4A–D** **)** possibly due to the pathogenic organisms from the orally administered sepsis feces. Accordingly, the kidney injury (mainly determined through renal tubular damage such as vacuolization, swelling, and desquamation) and liver damage (mainly evaluated by cell vacuolization) in CLP with sepsis feces were higher than in CLP with sham feces, while cell apoptosis (brown color stained in activated caspase 3 staining) in the spleen was not different between these groups **(** **Figures 4A–D** **)**. Different from the fecal gavage of sepsis feces (36, 52, 53), we explore the impacts of the transfers of fecal viral particles from sepsis feces. As such, oral gavage of fecal viral particles that separated from feces of sepsis mice attenuated several sepsis parameters, including kidney and liver injuries (serum creatinine and alanine transaminase), bacteremia, endotoxemia, gut barrier defect (FITC-dextran assay), and serum cytokines (TNF-α, IL-6, and IL-10), but not survival analysis **(** **Figures 5A–I** **)**.

**Figure 6:**
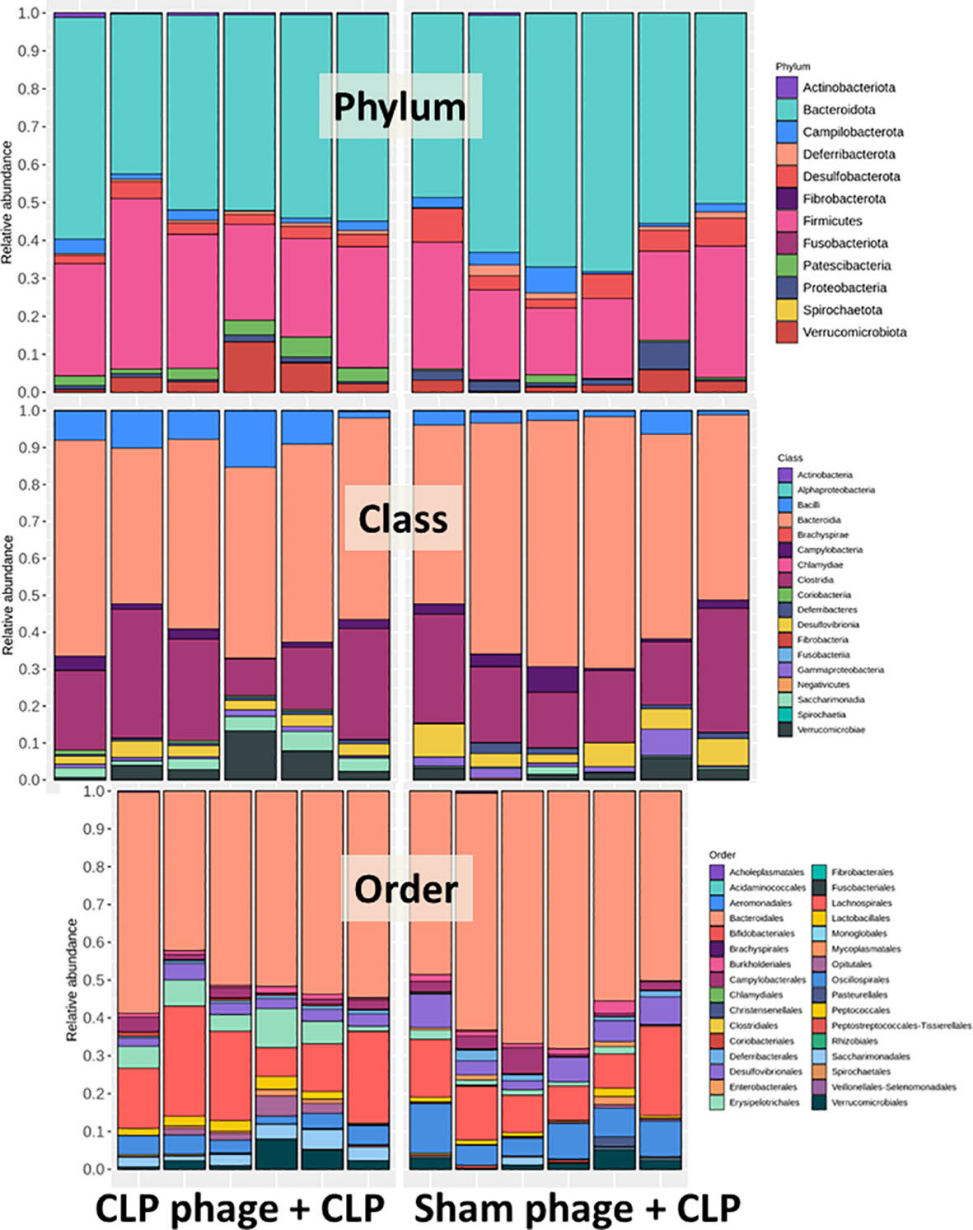
Fecal microbiota analysis from mice at 24 h after cecal ligation and puncture (CLP) surgery that was pre-conditioning by orally administration with the viral particles from sham (sham phage + CLP) or CLP mice (CLP phage + CLP) as determined by the relative abundance of bacterial diversity at phylum, class, and order levels of the analysis are demonstrated.

**Figure 7:**
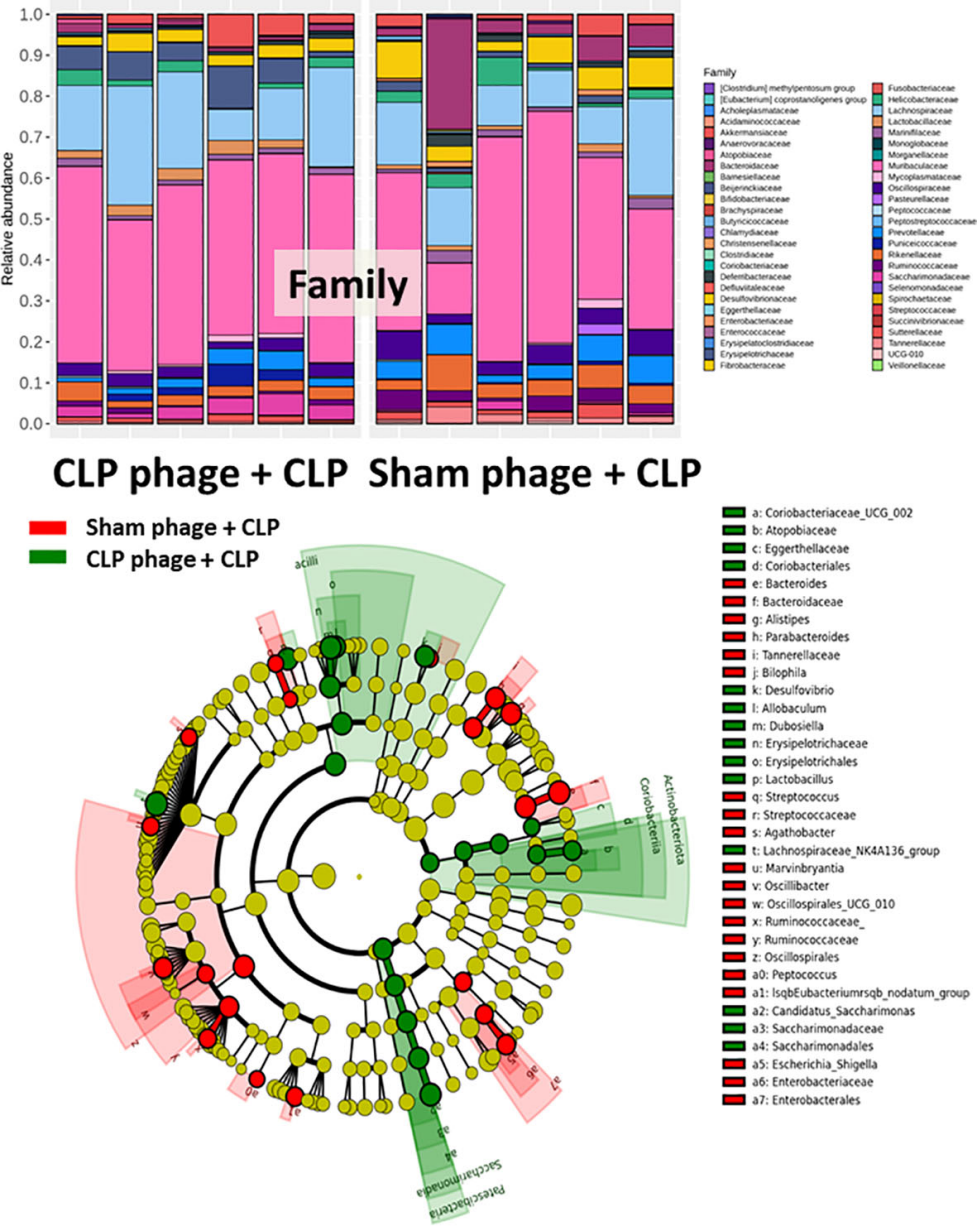
Fecal microbiota analysis from mice at 24 h after cecal ligation and puncture (CLP) surgery that were pre-conditioning by orally administration with the viral particles from sham (sham phage + CLP) or CLP mice (CLP phage + CLP) as determined by the relative abundance of bacterial diversity at the family level and cladogram plot are demonstrated.

**Figure 8:**
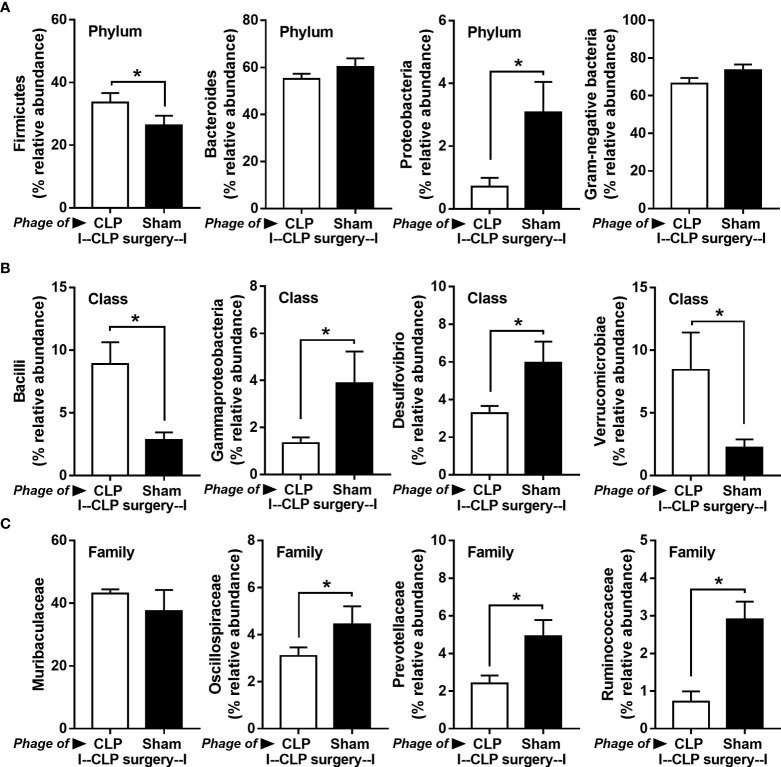
Fecal microbiota analysis from mice at 24 h after cecal ligation and puncture (CLP) surgery that were pre-conditioning by the oral administration with the viral particles from feces of sham (phage of sham) or CLP mice (phage of CLP) as indicated by graph presentation of the relative abundance of bacterial diversity for some important bacteria in phylum level (with total Gram-negative bacteria in feces) **(A)** and in class and family levels of the analysis **(B, C)** are demonstrated. *, *p* < 0.05 between the indicated group.

Because of the possible different influences of fecal bacteriophages between sham and CLP mice, bacteriome analysis in mouse feces at 24 h post-CLP after the 5 days of gavage by viral particles from sham and CLP mice was conducted **(** **Figures 6**, **7**, **8** **)**. Accordingly, viral particles from CLP reduced Proteobacteria (the pathogenic bacteria) and increased Firmicutes (the possible beneficial bacteria) (25), while they did not alter the abundance of *Bacteroides* (in phylum level of the analysis) and total fecal Gram-negative bacteria **(** **Figure 8A** **)**. In the class and family levels of analysis, CLP viral particles decreased pathogenic bacteria, such as Gammaproteobacteria and *Desulfovibrio* (54, 55), and increased some beneficial bacteria, such as Bacilli (including probiotics *Lactobacillus* spp.) and Verrucomicrobia (including *Akkermansia* spp. with the possible enhanced gut integrity) (56, 57) **(** **Figures 8B, C** **)**. However, we could not isolate phages from feces of both sham and sepsis mice using the culture with crude feces (aerobic and anaerobic processes) or the high abundance of bacteria that were isolated from feces (*Escherichia coli*, *Klebsiella pneumoniae*, and *Pseudomonas aeruginosa*) (data not shown). Perhaps, i) the beneficial phages are against several specific species of bacteria and ii) the combination of phages in the preparation of fecal viral particles, but not a single dominant phage is important for the sepsis attenuation effect..

## Discussion

Sepsis altered all organisms in the gut. The transfers of crude sepsis feces worsen sepsis, while the crude sham feces did not alter sepsis. However, the viral particles from sepsis feces, but not from sham feces, attenuated sepsis.

### The correlation among bacteria, fungi, and viruses in the gut during sepsis

The enhanced bacteria with possible pathogenicity, especially Proteobacteria, in the gut after sepsis is well-known as a result of either the local intestinal inflammation (such as *Clostridium* infection) or systemic responses (bacteremia with septicemia) (58). While some intestinal gut microorganisms directly decrease the beneficial bacteria (partly through the competition for nutrients), systemic infection indirectly alters the gut microbiome through several mechanisms, for example, an intestinal oxygen alteration (vasodilatation-induced hyperoxygenation or low blood pressure-induced hypoxia) and the select growth of some organisms from phospholipids (derived from the dying enterocytes) or the antibiotic peptides (59). Unfortunately, most of the bacteria with possible pathogenicity usually have more protecting factors against harsh microenvironments than beneficial bacteria (59). Regarding the gut fungi, Ascomycota in the phylum-level analysis was similarly predominant in the feces of both sham and sepsis mice. However, the abundance of *Myrothecium* in the genus-level analysis in sepsis feces was lower than in the sham group, which might partly initiate a selective environment for some microorganisms with possible pathogenicity. Because sepsis significantly reduced *Myrothecium*, which produces some molecules against several harmful factors to the host, including some organisms (60) and toxic substances from enterocytes (61, 62), the reduced *Myrothecium* might be another factor associated with sepsis-induced gut dysbiosis.

With gut virome analysis, there were more prominent *Myoviridae* (in sham) and *Podoviridae* (in CLP), the main components of several phage cocktails in other studies (48), without the significant differences in other viral families between sham and sepsis. Both *Myoviridae* and *Podoviridae* are phages against several *Staphylococci* spp., while only *Myoviridae* can attack coagulase-negative staphylococci (63), the gut bacteria causing neonatal sepsis (64). Because Proteobacteria was enhanced by sepsis and *Podoviridae* eliminated some Proteobacteria (such as *Citrobacter freundii*) (47), the enhanced *Podoviridae* in sepsis might be the phage against sepsis-induced gut bacteria with possible pathogenicity. Although these sepsis-induced viruses might have a potential for ameliorating septic inflammation or pathology, *Podoviridae* is a family of very short-non-contractile tail viruses in the order Caudovirales with 130 species in this family, which possibly attenuate several important sepsis-associated bacteria (*P. aeruginosa*, *E. coli*, *Salmonella* spp., and *Staphylococcus* spp.) (47, 48, 65, 66). The subsequent tests of the *Podoviridae* cocktail in sepsis are of interest. Nevertheless, there are still limited data on the non-bacterial gut organisms in sepsis compared to the larger volume of literature on sepsis-induced bacterial dysbiosis. Here, we hypothesize that CLP sepsis induces the harsh microenvironment leading to the overgrowth of Proteobacteria with the reduced beneficial organisms resulting in an increase in phages against Proteobacteria as a natural organismal control. Unfortunately, during sepsis, the improved beneficial phages are unable to keep up with the bacteria with possible pathogenicity, leading to increased gut dysbiosis and sepsis disease progression. It would be interesting to see further research on the interkingdom association of gut microbes in sepsis.

### The transfers of viral particles from sepsis feces, but not the crude feces, attenuated sepsis

The fecal transplantation of sepsis feces worsen sepsis, partly due to increased pathogenic bacteria and worsening of gut dysbiosis, supporting previous publications (36). Surprisingly, administration of viral particles from sepsis feces, but not from sham feces, attenuated sepsis as indicated by several parameters (kidney injury, liver damage, gut barrier defect, and serum cytokines). Despite the possibility of the transfer of other beneficial factors (such as short-chain fatty acids) from sepsis feces, the washing step during the preparation of viral particles (see *Materials and methods*) should decrease this contamination, and the experimental impacts might mainly be due to the viral particles. These data implied that there were higher beneficial phages in sepsis feces than in sham feces, possibly for natural control of the overgrowth of harmful microorganisms during sepsis. Although administration of viral particles from sepsis feces slightly decreased Firmicutes (the possible beneficial bacteria) (67), pathogenic Proteobacteria (68) were prominently reduced. Because phages infect only some specific bacteria that express similar molecules for viral entry (69, 70), the effects of viral particles on several bacteria imply the presence of multiple phages as the natural cocktails against pathogenic bacteria in sepsis feces. Although these beneficial phages might be the phages against both aerobes and anaerobes, the separation and identification of these phages from mouse feces are difficult. Indeed, we could not identify phages using either mixed or isolated bacteria (both aerobes and anaerobes) that were extracted from mouse feces (both sham and sepsis groups) (data not shown). Despite these limitations, isolation of viral particles against pathogenic bacteria from the infected host before the *ex vivo* phage propagation and re-administration of these natural phage cocktails into the host is an interesting hypothesis to test. With this strategy, the extensive preliminary preparation of phage cocktails and the pre-form test for phage bactericidal activity might not be necessary because the proper combination of phages is directly isolated from the host during the natural control of harmful microorganisms. More studies to test this hypothesis are warranted.

### Limitations and clinical translation

Because only a sepsis model induced by intra-abdominal infection using CLP surgery was used, the results might not refer to sepsis with other sources of infection (such as pneumonia or renal sepsis). Although there are several intra-abdominal sepsis models, including CLP, colon ascendens stent peritonitis (CASP), and cecal slurry injection (intraperitoneal injection of cecal contents from a donor rodent) (71), the CLP model was used here due to the possible less interference on the gut microbiota. While the foreign material of stent in CASP and the manipulation of feces from the rodent donor might interfere with the growth of some organisms, CLP seems to have less influence on gut microbiota.

**Figure 9:**
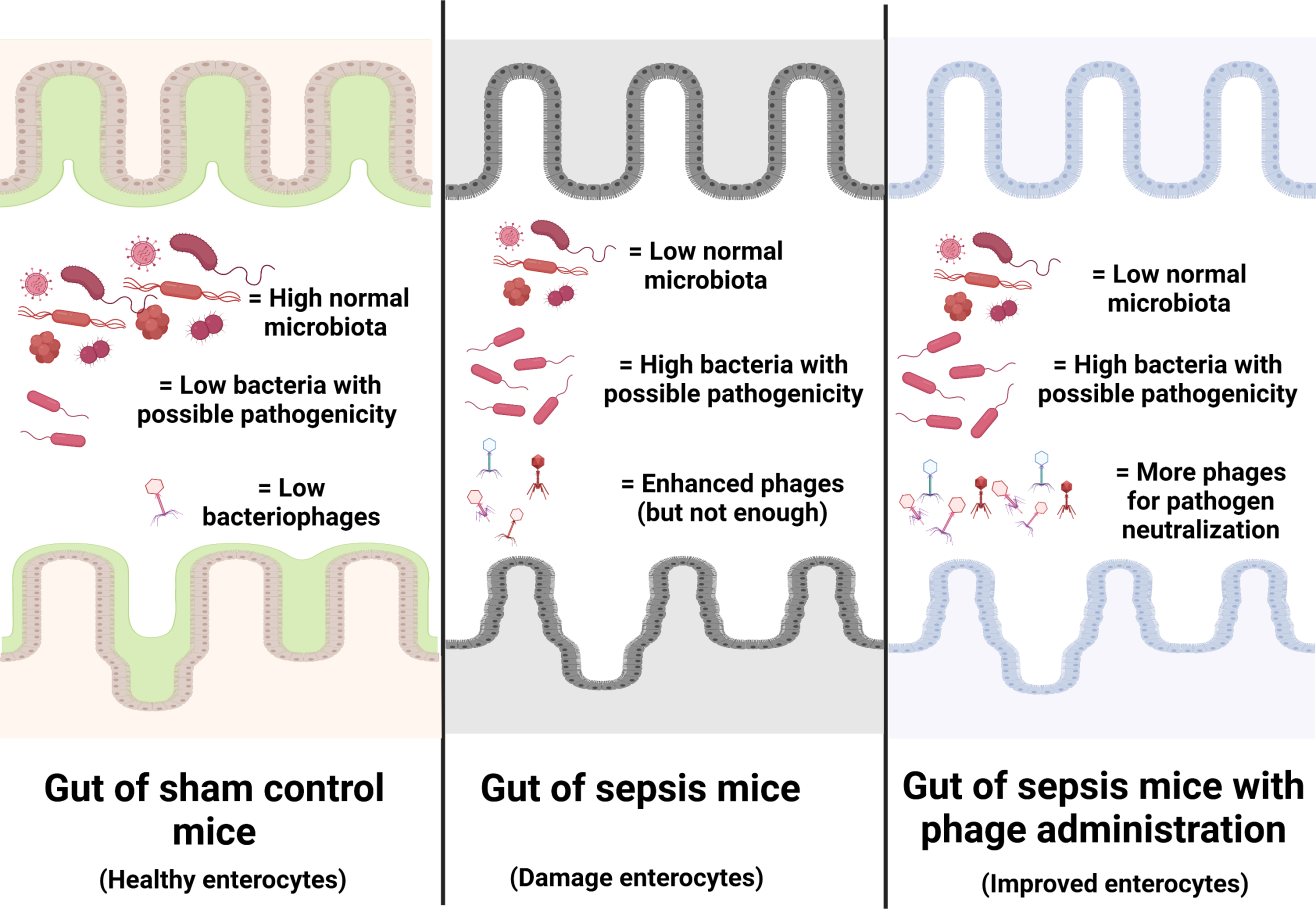
Illustration of the working hypothesis indicating gut dysbiosis (increase in bacteria with possible pathogenicity with decreased normal microbiota with possible enhanced bacteriophages) in the gut of sepsis mice when compared with sham mice. The administration of phages, isolated from other sepsis mice, increased phages to the level that is enough for the more effective neutralization of pathogenic bacteria. Phage isolation from the sepsis host with a rapid process of phage propagation before re-administration into the sepsis host is of interest for further test.

Due to the specificity of the individual type of phages that can infect only a limited type of bacterial species, the preparation of a combination of a large variety of phages against a single bacterial species as ‘phage cocktails’ is necessary (72). For example, anti-*Pseudomonas* phages that separated from a patient with *P. aeruginosa* infection might not be effective in another patient with *P. aeruginosa* infection (the same bacteria with a subtle difference), and then the anti-*Pseudomonas* phage cocktails extracted from several patients will be necessary for clinical use. Hence, a huge collection of phages, possibly with a very high cost and long duration of preparation, will be necessary for the ready-to-use phage cocktails for clinical situations. From our data, i) there were phages against endogenous bacteria in the gut, ii) there were alterations of endogenous phages along with the change in gut bacteria (toward pathogenic bacteria in sepsis), and iii) there was not enough quantity of the endogenous phages for the effective inhibition of pathogenic bacteria during sepsis (**Figure 9**). Hence, for the patients infected with the endogenous bacteria, the extraction of the naturally developed endogenous phages, before administration back to the host, might overcome the necessity for the preparation of phage cocktails that will reduce the cost of phage therapy. The rapid processes of extraction and propagation of the endogenous phages from the patients themselves might be interesting for the adjuvant therapy in sepsis. More studies are warranted.

In conclusion, CLP sepsis alters all organisms (bacteria, fungi, and viruses) in the gut, and the natural cocktails of phages that adapted against the altered bacteria might be beneficial for sepsis attenuation. The isolation of phages from the host with rapid *ex vivo* propagation before re-administration to the host is a proposed new strategy of sepsis adjuvant therapy.

## Data availability statement

The datasets presented in this study can be found in online repositories. The name of the repository and accession number can be found below: NCBI Sequence Read Archive; PRJNA838435

## Ethics statement

The animal care and use protocol (SST 010/2562) was certified by the Institutional Animal Care and Use Committee of Chulalongkorn University’s Faculty of Medicine in Bangkok, Thailand, in compliance with the US National Institutes of Health criteria.

## Author contributions

WC and AL conceptualized, designed, and managed the study. WC, NS, PV, VS, SC, SP, and AL performed the experiments. WC and AL performed the data analysis. WC and AL provided the key reagents. WC and AL wrote the manuscript. WC received the funding. AL, AS, and MS performed supervision. All authors made substantial contributions to subsequent versions of the manuscript and approved the final version.

## Funding

This research paper is supported by Specific League Funds from Mahidol University.

## Conflict of interest

The authors declare that the research was conducted in the absence of any commercial or financial relationships that could be construed as a potential conflict of interest.

## Publisher’s note

All claims expressed in this article are solely those of the authors and do not necessarily represent those of their affiliated organizations, or those of the publisher, the editors and the reviewers. Any product that may be evaluated in this article, or claim that may be made by its manufacturer, is not guaranteed or endorsed by the publisher.
